# Editorial: ELSI in Human Enhancement: What Distinguishes It From Therapy?

**DOI:** 10.3389/fgene.2020.00618

**Published:** 2020-06-23

**Authors:** Dov Greenbaum, Laura Y. Cabrera

**Affiliations:** ^1^Interdisciplinary Center Herzliya, Herzliya, Israel; ^2^Department of Molecular Biophysics and Biochemistry, Yale University, New Haven, NY, United States; ^3^Center for Ethics and Humanities in the Life Sciences, Department of Translational Neuroscience, Michigan State University, East Lansing, MI, United States

**Keywords:** enhancement, genetics, therapeutics, CRISPR, editorial

This ebook is timelier than we could have expected.

While this tome was envisaged more than a year ago, its publication unpredictably closely follows worldwide outrage at the news that two embryos, now children, were genetically enhanced via CRISPR, a genetic engineering technology, with a potential third child on the way.

Among the many justifiable (and some unjustifiable) concerns and considerations associated with this incident was an issue raised in many of the papers herein, and one that continues to confound scientific researchers and ethicists alike: What distinguishes scorned enhancement from celebrated therapy? Is there a clear line that can be drawn that makes one research path acceptable while the other is shunned?

Simplistically, enhancement in the biomedical context could be defined as intervention with the primary aim of overcoming those biological limitations that afflict the average person; these limitations may be inborn or acquired later in life. Some interventions are aimed to radically alter human biology, while others are more superficial.

In contrast, therapy is designed to help those who suffer from afflictions (not necessarily a trivially defined term, as shown in at least one of the papers in this compendium) that are not average, and result often in a standard of living that is below average. Succinctly: Enhancements seek to raise the user beyond the norm, whereas therapy seeks to bring the user up to the norm. A simple-minded example would be LASIK surgery. Eye surgery performed to bring a patient to 20/20 vision would be therapy, whereas surgery meant to provide super-human eyesight would be enhancement.

The LASIK example further importantly illustrates that it is rarely the technology itself that marks the difference between the two. Many factors associated with both therapy and enhancement are also often indistinguishable: Both therapeutics and enhancements can be implemented at any time in life: from pre-conception to near-death, and both can be accomplished via genetic manipulation, pharmaceutical, mechanical, biomechanical, and/or other methodologies. Both can be invasive or non-invasive, they can be permanent, semi-permanent, or temporary. In some instances, both therapies and enhancements can affect the germline of the individual, passing on the changes to their offspring.

Returning to the case of the aforementioned enhanced children, their embryos were altered to provide them with a rare genetic variant that could ostensibly provide some natural immunity against HIV infection. While arguably potentially therapeutic, most ethicists maintained that given the cheap and proven standards methods for preventing HIV infection, the resulting genetic manipulation was effectively more enhancement than therapeutic, and as such, abhorrent.

We can nearly fit this case into the above proposed definition of enhancement: Whereas the average person could use prophylactics, only the distinctive few, carry a helpful genetic variant. But, here is where the classification breaks down: the helpful genetic variant is by definition not superhuman, it exists in a not insubstantial portion of the population.

This ongoing discussion regarding therapy vs. enhancement is not simply academic: The rate of innovation in this area requires that legal jurisdictions decide what is, and what is not acceptable manipulation of the human body, perhaps sooner than we could have anticipated. In most legal jurisdictions, when restrictions are proposed for genetic manipulations, those restrictions typically will allow for therapeutic intervention, with regulators balking at the thought of enhancement, even when the exact same technology is employed. Thus, while there is no rule of thumb, in general, the closer interventions are to therapeutic goals, the easier they are to be regarded as acceptable. Further confounding this issue: there remain many enhancements, such as those pursued by athletes, military and other commercial industries that nevertheless often receive at least a grudging pass from the relevant regulatory bodies.

To some degree, there will likely never be a bright line distinction, and there will always be a visceral response to many areas of human enhancement technologies, even those that might seem to some as therapeutic: to paraphrase a US Supreme Court judge, many just know it when they see it. In these instances, the populist concerns tend to lean more toward issues related to playing God, not being natural, or how the enhancement somehow threatens an important aspect of the human condition, or simply some undefinable but palpable je ne sais quoi. These fuzzy feelings are legitimate but harder to deal with, particularly by the law. Whereas, other concerns that touch on issues of social justice, agency, regulation, as well as specific concerns attached to the specific populations using such interventions are much more manageable for the regulator.

And as we advance more innovative technologies, the stakes have clearly been raised regarding the many ethical, legal, and social concerns. And while we do not aim to solve this issue, this Frontiers Research Topic provides an overview on the ethical, social, and legal concerns raised by a variety of enhancement modalities, as well as different lenses on the topic from a broad spectrum of scholars in sociology, philosophy, genetics, neuroscience, and ethicists.

Several of the contributions to this e-book challenge other longstanding views in this area as well, and propose new frameworks oriented that can be helpful as we anticipate a lively and longstanding debate regarding human enhancement.

In the following, we provide a brief overview on the content of the e-book on “ELSI in human enhancement.”

The paper from Bruynseels et al. looks to the novel idea of incorporating the engineering concept described as Digital Twins. A digital twin is effectively a software version of the original, a computer model that is fed by numerous sensors that provide sufficient data via continuous monitoring to accurately reflect not only the architecture of the organic model (e.g., person) itself but also the real time dynamic of the original: a “data magnifying glass.”

According to the authors this is a feasible reality, given for example, the growing availability of a wide variety of wearable sensors. The authors argue that digital twins will be helpful in drawing a useful quantitative distinction between health and disease. This distinction should allow for a more nuanced appreciation of what is therapy and what is enhancement, where therapy relates to the maintenance or restoration to a clearly definable normal, that normal would likely be different in each individual. The predictive powers of such a system would also support therapeutics in asymptomatic individuals as while they are perceptively healthy, the digital twin would suggest otherwise.

The issue about how realistic are the scientific assumptions of the neuroenhancement debate is tackled by Schleim and Quednow. In particular, the authors suggest that all the hype notwithstanding we have yet to witness recent substantial innovation in the area of neuroenhancement drugs. The authors, noting the history of enhancement drugs are especially pessimistic regarding the near-future of this field. Moreover, they suggest that given the less than optimal state of the psychopharmacology field, resources are better used for the sick rather than the otherwise healthy.

Shook and Giordano address the vividly discussed area of moral bioenhancement for social welfare, but with a focus on whether or not civic institutions are ready for dealing with the consequences of such type of enhancement. They argue that if moral bioenhancement is to benefit both oneself and others it need to be conducted hand in hand with enhancement of local social conditions and civic institutions. They provide an hypothetical case of how the criminal justice system would deal with someone who has already received a civic enhancement, an enhancement which “would yield a large and reliable reduction in a person's behavior that could be threatening to other people, or would initiate and escalate violence.” Their conclusion is that civic institutions are ill prepared to handle such scenarios, and suggest that neuroethics can help develop answers by working with other disciplines.

Hyun introduces the novel discussion relating to what they call the genetics of ethnicity and diet. In particular this emerging trend could call into question the necessity of the scientific field of anti-doping, as well as highlighting the unintended cultural impacts of this field. Particularly Hyun is concerned with how genetic research in the area of antidoping can have unintended consequences such as the facilitation of uninformed discussions on genetic determination and racism in sports.

So et al. provide one of the most prescient submissions in this collection, focusing specifically on a central question regarding the recent genetic manipulation of three embryos by He Jiankui. As described above, Dr. He ignited an international firestorm when he announced the birth of twin girls, Lulu and Nana who had been modified and possibly enhanced. The two girls, and possibly a third, had their genomes edited with the goal of deactivating the CCR5 gene.

While the twin's father was HIV positive, neither girl was at risk for contracting HIV and as such, many had argued that the genetic modification was an enhancement, not a therapy, even though the results could be construed as providing a therapeutic outcome.

Some of the discussion regarding Dr. He also focused on an issue, not necessarily touched upon in this collection of papers, but nevertheless highly relevant to human enhancement efforts: What about the externalities? The girls' modified CCR5 gene does not confer 100% resistance, and whatever resistance it does confer comes at a price in the form of increased susceptibility to West Nile Virus and the Flu. Moreover, the data suggests a further important caveat, while the intent was to incorporate the delta 32 variant into the girls' genomes, the data suggests that neither girl received that particular known variant: Lulu has a different mutation and Nana has two separate mutations.

In the meantime the girls currently appear to be healthy, however the inexactness of the science would further suggest that these sorts of manipulations be limited to only instances where the health of the child is clearly at risk, not for seemingly trivial enhancements. A further externality of the case, the growing global consensus for a moratorium that could also limit innovation in this area, a moratorium that might have been perceived to be unnecessary had He employed the technology for a clearly therapeutic purpose.

Further, as the authors of this paper point correctly predicted, the use of germline modification by He has led to an outcry against this type of modification and has led to the likelihood that now most efforts to provide resistance to communicable disease (RCD) will be reflexively labeled enhancements and not therapeutics. This paper will continue to be relevant both as the He scandal continues to play out, and likely long after.

Cabrera discusses the ethical importance to reframe human enhancement from its individual-based orientation and reductionist approach to a more inclusive and population-oriented one. She argues that lessons can be learnt from a population health perspective to focus on addressing environmental factors, instead of just individual ones, in order to attain optimal performance and well-ness of individuals at the scale of populations. Cabrera argues that this reframing of enhancement, together with the focus on equitable and accessible interventions, can also be regarded as a reasonable path in addressing social inequalities.

A novel perspective on neuro-enhancement is provided by the “Neuro-Enhancement Practices Across the Lifecourse: Exploring the Roles of Relationality and Individualism” from O'Connor and Nagel. They also argue that relationality, rather than pure individualism, may be a more suitable framework for conceptualizing findings in the empirical literature about everyday engagements with neuroenhancement. The authors focus on two major areas within the neuroenhancement discourse, (1) enhancing children's brains, and (2) preventing age-related cognitive deterioration. Readers gain an insight into how those concerns are essentially relational, and how they shape the ways in which neuroenhancement concepts and technologies unfold in everyday life.

Finally, the contribution of Tamir focuses on considerations of children rights to be genetically enhanced. Like So et al. forewarning submission speaks to one of the issues associated with the aforementioned Dr. He's research, specifically the lack of consent by the children, and their subsequent children regarding the germline genetic modification of the CCR5 gene. Although Tamir refers specifically to the post-natal phase of a child's life, where the lack of consent is most glaring given that the children would actually have a voice, many of the issues are particularly relevant even today, at least to the to the very small subset of individuals who have already undergone prenatal genetic enhancement. A subset that will likely soon grow.

In fact, the two conditions that Tamir sets out as necessary for the wide introduction of post-natal genetic enhancements, are precisely the areas where Dr. He failed in his efforts: The CCR5 gene in question was not perfectly targeted, the children did not obtain the desired delta 32 allele, and the children's genomes were not efficiently modified. At least one of the girls is heterozygous for the desired allele.

It is unlikely that even the uniform outcry associated with Dr. He's work will not prevent future enhancement efforts on both prenatal as well as eventually post-natal children. As such, Tamir's efforts to create a right for children to be or not be genetically enhanced is an important and valuable effort.

Overall, the contributions that form this eBook on ethical legal and societal implications in human enhancement demonstrate the variety of concerns and modalities involve in the quest to enhance humans. It also suggests the importance of inputs coming from regulatory and legal mindset when considering human enhancement, as many of the relevant issues and determinations would benefit from sharp and clear legal definitions and distinctions. We are encouraged to see that researchers from many different disciplines brought different insights into the discussion around human enhancement.

## Author Contributions

All authors listed have made a substantial, direct and intellectual contribution to the work, and approved it for publication.

## Conflict of Interest

The authors declare that the research was conducted in the absence of any commercial or financial relationships that could be construed as a potential conflict of interest.

